# Constipation in Childhood

**Published:** 1906-04

**Authors:** E. S. McKee

**Affiliations:** Cincinnati, Clinician to Gynecological Department Medical College of Ohio. University of Cincinnati


### Constipation in Childhood.

By E. S. McKEE, M. D., Cincinnati,
Clinician to Gynecological Department Medical College of Ohio. University of Cincinnati.

IN children the indications should be met as far as possible by hygienic surroundings. Young children should be trained from their earliest years to regular habits. A regular hour, if possible directly after breakfast, should be selected when probably 15 minutes should be devoted to the securing of a proper evacuation. This visit should be made daily at the same hour with religious regularity whether the desire be felt or not and in time the trials will become more and more successful. The water closet should be made comfortable enough so far as the temperature, rugs, pictures, odors and accessibility are concerned, that he required amount of time can be spent pleasantly and comfortably without danger of contracting cold. Inaccsssible, cold and distant privies such as are found in the country and in old suburban houses, are a fertile source of constipation.

A stool having been apparently finished a few minutes should be allowed so that a residue of fecal matter which may remain in the bowels may find its way into the rectum and be expelled. Else it remains there for perhaps 24 hours, blunts the sensibility of the rectum, thus delaying a cure. A call of nature, no matter when it occurs should be obeyed. This is sometimes easier in theory than in practice. The squatting posture (out behind the barn or in the fence corner) nature's position, by giving the fullest play to the muscles often enables the person to successfully evacuate the bowels when a seat on the ordinary water closet is unsuccessful.

In children who sit on the seat of the water closet for adults, their feet dangling in the air, the muscular force is very seriously abridged and they should have low seats where their feet would reach the floor. Really the best water closet is where the occupant squats on the floor or on a beam thus giving each and every muscle full play.

Diet is of the greatest importance. Fruit is usually of benefit, such as figs, dates, prunes, especially if stewed; so also are baked apples. Oat meal is beneficial to most persons. Brown bread and molasses are anticonstipating. The bread should be made of whole wheat. Milk should often be supplanted by cream. Berries, that is the seeds, are usually constipating. Water, hot or cold, taken an hour before breakfast is beneficial. The sparkling waters, vichy or apollinaris, are often better than the plain ones.

Massage is useful in conjunction with other means but it is rarely successful alone. It should occupy 8 or 10 minutes before rising and after retiring. The hand should be warm. The object is to give a rotary motion. The hand or finger tips are fixed on the abdominal wall and by means of this the underlying intestines are to be kneaded.. There should be no friction of the hand upon the skin but of the abdominal wall upon the intestine. This should be a circular motion and should commence in the patient's lower right quadrant of the abdomen (ileo-cecal valve) extend up, across and down the abdomen following the course of the colon. As massage has been very justly described as exercise without effort, it follows that exercise is a very beneficial thing where constipation is present.

Suppositories of soap or glycerine are good but should not be used too often or for too long a time. A good combination for a suppository to be used night and morning for a considerable time is the following: ℞ Aloin 0.03 or gr., ss. ext. belladonnæ 0.03 or gr ss., ext. nucis vomicæ 0.06 or gr 1.,01. theobromæ 8.00 or dram II. Misce et fiat suppositoria no xii.

Enemata are good for temporary relief. Hard impactions of feces may be broken up and expelled by injections of an ounce of sweet oil or larger injections of soap and water. One teaspoonful of glycerine to one tablespoonful of water is very effective.

*Internal Medication.* Calomel is indicated in dry white stools with much gas. Use ¼ to ½ grain tablets every night for three or four nights followed by salts or seidlitz powder if bowels do not act in the morning. Cascara may be given in the form of the elixir, one-half to one teaspoonful, or the fluid extract 1 to 5 drops. For long continued use phosphate of soda (granular) ⅓ teaspoonful in water one to three times a day may be advised. Preparations of malt are slightly laxative and also nutritious. Frequent use of small quantities of olive oil mixed in the food of a child is an excellent plan.

For a child two or three years old with chronic constipation I would give: massage 8 to 10 minutes morning and evening, juice of half an orange and a glass of water or vichy immediately on rising. For breakfast I would advise oat meal with cream, dried bread and butter, one egg, half a glass of milk, with cream and water added; for dinner, soup, one starchy vegetable, for example, potato with cream, and one green vegetable, beefsteak, baked apple, prunes, dried bread and butter, and water; for supper, cream toast, one egg, dried bread and butter or graham crackers, half a glass of milk with cream and water added and suppositories of aloin, etc., at bed hour. The well known aloin, strychnia, and belladonna comp. pill divided into pills of one-fifth the ordinary strength are very valuable in the treatment of constipation in children, repeating the pill one, two or three times a day as necessary.

In the constipation of sucklings a change in the diet of the mother may be tried, or from one to three teaspoonfuls of cream may be given before each nursing. In artificially fed children the top milk with cream should be fed. Water, barley water, or oat meal will sometimes obviate the difficulty. As laxatives, simple syrup, manna, olive oil, castor oil, or fluid magnesia may be sufficient. A conical piece of soap inserted into the rectum is sometimes sufficient as is even a thermometer if inserted at regular intervals. In infants after the fifth or sixth month costiveness is an indication for the introduction of starchy matter into the diet.

The daily injection in infants of warm soap suds by means of a soft bulbed ear syringe, or of glycerine gtt xv-xx in a teaspoonful of water is very successful. The following may be given: ℞ Calcined magnesia, (Magnesium Oxide), Sacch. Lactis aa 0.50 or gr vii ss. Put a piece of flaked manna in each bottle of artificially fed infants food. Ten drops of syrup of figs, 2-4 drops of fluid extract of cascara sagrada, a pinch of salt in the bottle, the addition of Mellin's food to the diet, the twice daily massage, the addition to each bottle of milk of 2-4 gr phosphate of soda, an increase in the proportion of cream, Tarrants seltzer aperient, 10 grains in the milk, a little milk of magnesia added to the milk or water—these are usually successful, one or all, used in consecutive order.

10 West Seventh Street.

